# The Absolute Tests of Death

**Published:** 1889-11

**Authors:** 


### THE ABSOLUTE TESTS OF DEATH.

The proofs of absolute death are eleven in number :—

*Cessation of Respiration Test.*—The first of these signs of death,—the cessation of the indications of respiratory function, — although useful in a general sense, is not by any means reliable. The old breath test,—

> "If that a feather move across the breath,
> Then life remains,"—

is too fallacious to be of real service, as is also the common mirror-test, a passive exhalation of water being quite sufficient to produce a deposit of moisture upon the bright reflector, even when life may be quite extinct.

*Cardiac and Arterial Failure Test* —Of the two signs—the pulse and the sounds of the heart—the pulse is the more important.

*Venous Turgescence Test.*—I consider the vein-pressure proof one of the very best and readiest of all. It is carried out by placing a hard substance, like a bit of mill-board, on the forepart of the wrist, so as to prevent pressure on the radial and ulnar arteries ; then a fillet is tied firmly around the wrist, so as to compress the veins at the back of the hand. If upon this the veins, after a time, fail to enlarge, there is *prima-facia* evidence that no circulation is going on, and that life is certainly extinct. While this test alone is not sufficient to disprove the fact of life, it is yet one least of all likely to deceive.

*Reduction of Temperature Test.*—Reduction of the temperature of the body below the natural standard is a good test, but one that requires to be used judiciously. A reduction of a few degrees is not sufficient, since recovery after seven degrees of reduction has been known to take place. But if the temperature of the body found in the cavities, like the mouth, is below the temperature of the surrounding air, or if it even be reduced to 80°, the evidence is strong that life does not remain.

*Rigor Mortis Test.*—The existence of well-marked *rigor mortis* is one of the most certain proofs of absolute death.

*Coagulation of Blood Test.*—The condition of the blood in the veins, whether it be coagulated or not, is a most important matter. The act of coagulation is practically the same as that of *rigor mortis*, and when the two signs are present together, there can be no doubt of the fact of death.

*Putrefactive Decomposition Test.*—Of putrefactive decomposition as proof of death, little doubt is ever entertained ; but while in its slightest development it cannot be accepted as conclusive, there are certain decompositions that are definite. Whenever decomposition of the eyeball is pronounced, with shrinking of the ball and opacity of the cornea, it may be considered convincing proof of death.

*Strong Light Test.*—To the test made with strong light, in order to ascertain if in semi-transparent parts, like the hands, there is still redness of tint, I attach secondary importance.

*Electric Stimulus Test.*—I attach considerable importance to the excitation of muscular contraction under electric stimulus. It is a test which is often urgently called for, and it has its own value. A small battery for the Faradic current, a couple of long needles to attach to the electrodes, and two sponges are required. The muscles of the fore-arm are most convenient for testing, the needles being pressed into the muscles deeply.

*The Ammonia Test.*—To the hypodermic ammonia test, suggested by Montiverdi, I attach great importance. It is merely necessary in carrying it out to inject thirty minims of ammonia solution of sp. gr. 891° under the skin. If there still be a circulation through the part receiving the ammonia, there will be a reaction in the form of a blotch of a red erythematous color, wine red, with raised spots on the surface. If death has taken place, instead of a red blotch there will be a blotch of a dirty skin color, without a trace of red spots, which Montiverdi affirms is the only criterion of actual death known at the present time.

*The Bright Steel Test.*—The oxidation, or bright steel test of Cloquet and Laborde, has the advantage of being simple and physiological, and may be used as corroborative of other tests, if nothing more. A bright steel needle may be thrust into the biceps, left there in position a short time, then removed and put in a dry place. If it remains bright, it has plainly pierced a dead tissue. From the nature of the circumstances, the time for this test must be limited to a very short period after the supposed death.

Summary.—The following are the salient points of practice to be observed in cases where proofs of absolute death are demanded :—

Assuming that the respiratory functions fail to give evidence of life ; assuming that there is no arterial pulse or sign of cardiac motion ; assuming that there is an absence of *rigor mortis*, and that there are some remaining appearances of expression, of color or of warmth, which suggest the idea of possible life at low cardiac pressure, the practitioner will apply—

1. The Venous Turgescence Test.
2. The Coagulation of Blood Test.
3. The Electric Stimulus Test.
4. The Ammonia Test.

One further point of practice should still be carried out. The body should be kept in a room the temperature of which has been raised to a heat of 84° Fahrenheit, with moisture diffused through the air, and in this warm and moist atmosphere it should remain until destinct indications of putrefactive decomposition have set in. This final practice combines several practical advantages: It gives a favorable chance to restoration if so be the vital fire is not out, and if death has really occurred, it favors the development of *rigor mortis*, besides enabling the surgeon to carry out his responsible task in cases where he is not allowed to touch the dead, a condition not uncommonly enforced by members of the Jewish and other religious communities.—*Good Health.*

